# Editorial: Bioinformatics analysis of single cell sequencing and multi-omics in the aging and age-associated diseases

**DOI:** 10.3389/fnagi.2024.1384586

**Published:** 2024-03-07

**Authors:** Shouneng Peng, Ailan Wang, Junjun Ding, Lilach Soreq

**Affiliations:** ^1^Department of Genetics and Genomic Sciences, Icahn School of Medicine at Mount Sinai, New York, NY, United States; ^2^Beijing Hotgen Biotech Co., Ltd., Beijing, China; ^3^Zhongshan School of Medicine, Sun Yat-sen University, Guangzhou, China; ^4^Department of Molecular Neuroscience, Institute of Neurology, University College London, London, United Kingdom

**Keywords:** bioinformatics and computational biology, single cell sequencing, multi-omics, brain aging, neurodegenerative diseases (ND), Alzheimer's disease (AD), Parkinson's disease (PD)

Recognizing age as a pivotal risk factor for age-associated diseases, particularly neurodegenerative diseases (ND), such as Alzheimer's disease (AD) (Peng et al., 2021) and Parkinson's disease (PD), underscores the urgency of addressing age-associated disease challenges since life spans continue to increase. To achieve this, a thorough bioinformatics analysis encompassing human genomic, epigenetic, transcriptomic, proteomic, and metabolic modifications across normal aging and the onset and progression of diseases is imperative. This approach is crucial for gaining insights into the intricate mechanisms underlying age-associated diseases and for developing effective strategies to enhance the health and wellbeing of older individuals (Patel et al., 2019; Yang et al., 2020; Peng et al., 2021). Recent breakthroughs in cell type and single-cell research (Soreq et al., 2017, 2023) have unveiled the intricate molecular diversity within cells, shedding light on cell type-specific aging markers crucial for understanding brain aging and ND.

Exploring the intricate landscape of brain aging and ND, this Research Topic extensively examines multi-omics, cell type, single-cell, and novel algorithmic methodologies. A total of 10 articles enrich this exploration, each providing distinctive perspectives and valuable insights into the complex interplay of factors influencing these processes. In their comprehensive investigation of multi-omics data, Hao et al. meticulously analyzed copy number variation (CNV) profiles, transcriptomic data, and meta-information. The outcome of their study revealed a CNV-Gene-AOD (age of death) causality network involving three CNVs (DEL5006, mCNV14192, and DUP42180) and seven genes (*PLGRKT, TLR1, PLAU, CALB2, SYTL2, OTOF*, and *NT5DC1*) that play a role in regulating AOD in AD patients, irrespective of the severity of their AD condition. Notably, the researchers unveiled the significance of the plasminogen activation system (PAS) in influencing the longevity of AD patients. The findings provide valuable insights into the intricate molecular mechanisms associated with AOD in AD patients, shedding light on potential therapeutic targets and pathways for further exploration. Chen et al. revealed a notable sex-specific pattern, with exercise significantly reducing *GPNMB* cg17274742 methylation levels in males but not in females. This suggests a nuanced epigenetic regulation of *GPNMB* expression and the complex relationship between lifestyle interventions, epigenetics, and ND.

Lu et al. conducted a comprehensive metabolomics analysis identified 69 differentially abundant metabolites, with notable changes in amino acids, lipids, and microbiota-derived metabolites. Transcriptomic analysis further unveiled 376 differentially expressed genes in the aged hippocampus. The integration of multi-omics data from 35 differentially abundant metabolites and 119 differentially expressed genes for construction of a co-expression network illuminated changes in pathways associated with inflammation, microglia (MG) activation, synapse function, cell death, cellular/tissue homeostasis, and metabolism within the aging hippocampus. The study sheds light on potential targets for interventions aiming to mitigate age-related cognitive decline. Sang et al. conducted a comprehensive analysis employing metabolomics and proteomics, revealing notable reductions in the levels of key enzymes—pyruvate dehydrogenase complex, 2-oxoglutarate dehydrogenase complex, isocitrate dehydrogenase, and succinyl-CoA synthetase—in the brains of individuals with sporadic Alzheimer's disease (sAD) compared to controls. The study suggests that a deficiency in pantothenate in sAD brains may adversely affect energy metabolism, leading to mitochondrial dysfunction, oxidative stress, and impaired neurotransmission. This challenges the traditional emphasis on amyloid-beta and tau proteins, proposing metabolic anomalies as potential causative factors in AD. The findings highlight the importance of considering broader metabolic perspectives in understanding the underlying mechanisms of sAD.

Within our carefully curated compilation of cell type and single-cell research, Liu et al. present compelling evidence highlighting the role of MG in regulating wake/sleep cycles, demonstrating that replenishing MG can enhance stable wakefulness in aging mice. Simultaneously, Zhao et al. contribute valuable insights into the neurovascular unit (NVU) function, particularly regarding cellular senescence in MG and oligodendrocytes among AD patients. Additionally, they identify 15 key aging-related genes (AGs) that differentiate AD from normal controls. Niu et al.'s comprehensive investigation, leveraging single-nucleus RNA sequencing and plasma proteomics, identifies 12 differential genes/proteins associated with brain aging. Furthermore, they identify 93 cell-specific genes correlating with age, highlighting five plasma biomarkers (DSCAM, CNTN2, IL1RAPL2, CA10, GPC5) linked to brain aging.

These studies collectively underscore the significance of multi-omics and single-cell in uncovering biomarkers, disease genes, and potential drug targets for brain aging and ND. Additionally, three articles in our Research Topic employ machine learning to enhance biomarker discovery, predict disease genes, and explore drug-target interactions for providing valuable therapeutic insights in the realm of brain aging and ND. For example, Zou et al. selects eight feature genes (*ATP2B3, BDNF, DVL2, ITGA10, SLC6A12, SMAD4, SST*, and *TPI1*) by the LASSO model and spotlights the regulation of the gene *SLC6A12* by miR-3176, highlighting its strong correlation with dendritic cells and plasmacytoid dendritic cells in AD patients. This novel finding suggests its potential as a biomarker for AD, emphasizing its link to immune cells. In another study, Ma et al. introduces a network embedding method PSNE (preserving structure network embedding) to predict disease genes, successfully applying it to identify potential pathogenic genes for AD and PD. Zhou et al. introduces a new method for new drug target identification and drug repositioning in ND such as AD and PD. These innovative approaches collectively contribute to advancing our understanding and potential interventions for brain aging and neurodegenerative conditions.

Overall, these studies collectively propel our comprehension of the intricate relationships between cellular and molecular processes in brain aging and ND. The combining multi-omics, cell types, single-cell sequencing, and machine learning algorithms not only paves the way for novel avenues in biomarker discovery, disease gene identification, and potential therapeutic targets but also underscore the profound promise in enhancing our insights into brain aging and neurodegeneration.

Significantly, the noteworthy developments from this Research Topic should be stem cell aging, unveiling critical insights into age-dependent glial and endothelial impairments, particularly in the context of blood-brain barrier (BBB) and NVU dysfunction. The impairment of regenerative capacity in endothelial cells and glial cells stands out as a significant contributor to BBB dysfunction during aging. Additionally, triggering MG repopulation (gliogenesis, Liu et al.) (Elmore et al., 2018) emerges as a pivotal strategy and leading to the reversal of age-associated changes in neuronal gene expression, underlining the potential therapeutic impact of addressing stem cell aging in the context of brain aging and ND.

## Author contributions

SP: Writing – original draft, Writing – review & editing. AW: Writing – review & editing. JD: Writing – review & editing. LS: Writing – review & editing.
